# A Surrogate Animal Model for Screening of Ebola and Marburg Glycoprotein-Targeting Drugs Using Pseudotyped Vesicular Stomatitis Viruses

**DOI:** 10.3390/v12090923

**Published:** 2020-08-22

**Authors:** Takeshi Saito, Junki Maruyama, Noriyo Nagata, Mao Isono, Kosuke Okuya, Yoshihiro Takadate, Yurie Kida, Hiroko Miyamoto, Akina Mori-Kajihara, Takanari Hattori, Wakako Furuyama, Shinya Ogawa, Shigeru Iida, Ayato Takada

**Affiliations:** 1 Division of Global Epidemiology, Research Center for Zoonosis Control, Hokkaido University, Sapporo 001-0020, Japan; t.saito@czc.hokudai.ac.jp (T.S.); misono@czc.hokudai.ac.jp (M.I.); kokuya@czc.hokudai.ac.jp (K.O.); ytakadate@czc.hokudai.ac.jp (Y.T.); yurie-kida@czc.hokudai.ac.jp (Y.K.); hirom@czc.hokudai.ac.jp (H.M.); akinam@czc.hokudai.ac.jp (A.M.-K.); hattoritakanari@czc.hokudai.ac.jp (T.H.); 2 Department of Pathology, The University of Texas Medical Branch, Galveston, TX 77555, USA; jumaruya@utmb.edu; 3 Department of Pathology, National Institute of Infectious Diseases, Tokyo 208-0011, Japan; nnagata@nih.go.jp; 4 Laboratory of Virology, Division of Intramural Research, National Institute of Allergy and Infectious Diseases, National Institutes of Health, Rocky Mountain Laboratories, Hamilton, MT 59840, USA; wakako.asada@nih.gov; 5 Tokyo Research Park, Kyowa Kirin Co., Ltd., Tokyo 194-0023, Japan; shinya.ogawa.y5@kyowakirin.com (S.O.); shigeru.iida.6h@kyowakirin.com (S.I.); 6 Global Station for Zoonosis Control, Global Institution for Collaborative Research and Education, Hokkaido University, Sapporo 001-0020, Japan

**Keywords:** Filovirus, Ebola virus, Marburg virus, recombinant vesicular stomatitis virus, Syrian hamster, animal model, drug screening

## Abstract

Filoviruses, including Ebola virus (EBOV) and Marburg virus (MARV), cause severe hemorrhagic fever in humans and nonhuman primates with high mortality rates. There is no approved therapy against these deadly viruses. Antiviral drug development has been hampered by the requirement of a biosafety level (BSL)-4 facility to handle infectious EBOV and MARV because of their high pathogenicity to humans. In this study, we aimed to establish a surrogate animal model that can be used for anti-EBOV and -MARV drug screening under BSL-2 conditions by focusing on the replication-competent recombinant vesicular stomatitis virus (rVSV) pseudotyped with the envelope glycoprotein (GP) of EBOV (rVSV/EBOV) and MARV (rVSV/MARV), which has been investigated as vaccine candidates and thus widely used in BSL-2 laboratories. We first inoculated mice, rats, and hamsters intraperitoneally with rVSV/EBOV and found that only hamsters showed disease signs and succumbed within 4 days post-infection. Infection with rVSV/MARV also caused lethal infection in hamsters. Both rVSV/EBOV and rVSV/MARV were detected at high titers in multiple organs including the liver, spleen, kidney, and lungs of infected hamsters, indicating acute and systemic infection resulting in fatal outcomes. Therapeutic effects of passive immunization with an anti-EBOV neutralizing antibody were specifically observed in rVSV/EBOV-infected hamsters. Thus, this animal model is expected to be a useful tool to facilitate in vivo screening of anti-filovirus drugs targeting the GP molecule.

## 1. Introduction

The family *Filoviridae* includes five genera: *Ebolavirus*, *Marburgvirus*, *Cuevavirus*, *Striavirus*, and *Thamnovirus*. The *Ebolavirus* genus contains five species—*Zaire ebolavirus* represented by Ebola virus (EBOV), *Sudan ebolavirus*, *Taï Forest ebolavirus*, *Bundibugyo ebolavirus*, and *Reston ebolavirus*. The *Marburgvirus* genus consists of only one species, Marburg marburgvirus including the prototype filovirus, Marburg virus (MARV) [1]. The filovirus genome encodes seven structural proteins, an envelope glycoprotein (GP), a nucleoprotein, viral proteins (VP24, VP30, VP35, and VP40), and L protein. GP is the only viral surface protein of filoviruses and mediates attachment of virus particles to host cells and fusion of the viral envelope with the host cell membrane, which is the initial step of virus infection [2,3,4].

Except for Reston virus, members of genera *Ebolavirus* and *Marburgvirus* are known to cause severe hemorrhagic fever in humans and nonhuman primates (NHPs) with high mortality rates. The diseases caused by EBOV and MARV are known as Ebola virus disease (EVD) and Marburg virus disease (MVD), respectively [2,5,6]. Animal models of EVD and MVD have been developed in mice, rats, guinea pigs, hamsters, ferrets, and NHPs [7,8,9,10,11]. Of these, EBOV and MARV infections of NHPs are the gold-standard models because they best represent similar pathogenesis of EVD or MVD in humans [3,11]. Thus, the NHP model has been used for experimental studies of EBOV and MARV pathogenicity and for the development of vaccines and antiviral drugs [12,13,14,15,16]. However, the use of NHPs in a high-biocontainment laboratory is associated with high cost and technical difficulties; hence, some rodent models have been developed to deal with these problems [9,17,18,19,20]. 

Since wild-type EBOV (WT-EBOV) and MARV do not cause fatal disease in immunocompetent mice and guinea pigs, mouse-adapted EBOV (MA-EBOV) and MARV, and guinea-pig adapted EBOV and MARV, all of which cause lethal infection uniformly in these animals, have been generated by serial passage of the viruses [19,21,22,23,24]. Immunocompromised mice (IFN-α/β receptor-knockout (*IFNAR^-/-^*) and transcription factor STAT1-knockout (*STAT1^-/-^*) mice) are also used as alternative models since they are highly susceptible to WT-EBOV [25]. It has also been shown that infection of Syrian hamsters with MA-EBOV is a good model that gives increased coagulopathy, which is not generally observed in the other rodent models [26]. However, due to the requirement of BSL-4 facilities for handling infectious EBOV and MARV, the currently available animal models have limitations of their use for early-stage screening of anti-EBOV and MARV drugs.

In this study, we evaluated replication-competent recombinant vesicular stomatitis viruses pseudotyped with EBOV and MARV GPs (rVSV/EBOV and rVSV/MARV, respectively) to determine their utility as surrogate viruses for in vivo drug screening under BSL-2 conditions [27,28]. These viruses were shown to be highly potent live attenuated vaccine candidates against EVD and MVD in rodent and NHP models [29,30] and the rVSV/EBOV-based vaccine has been clinically approved in the EU and USA [31,32]. On the other hand, rVSV/EBOV was reported to cause lethal infection in *IFNAR^-/-^* or *STAT1^-/-^* mice and neonatal mice [33], suggesting its potential to cause disease in some animals. Here, we show that intraperitoneal inoculation of Syrian hamsters with rVSV/EBOV and rVSV/MARV induces acute and fatal infection and that rVSV/EBOV-infected animals can be completely protected by passive immunization with a neutralizing antibody specific to EBOV GP. These rVSV/EBOV- and rVSV/MARV-infected Syrian hamster models may be useful for in vivo screening of anti-filovirus drugs that target the GP functions.

## 2. Materials and Methods

### 2.1. Cells and Viruses

Vero E6 cells were grown in Dulbecco’s modified Eagle’s medium (DMEM) (Sigma) supplemented with 10% fetal calf serum (FCS) (Cell Culture Bioscience), 100 U/mL penicillin, and 0.1 mg/mL streptomycin (Gibco). Replication-competent recombinant VSVs (rVSV/VSV) (Indiana) and rVSV pseudotyped with EBOV (Mayinga) and MARV (Angola) GPs (rVSV/EBOV and rVSV/MARV, respectively) were generated as described previously [27]. rVSV/VSV, rVSV/EBOV, and rVSV/MARV were propagated in Vero E6 cells and stored at −80 °C until use. Virus titers were determined by a plaque assay and represented as plaque-forming units (PFU).

### 2.2. Monoclonal Antibodies

Based on the nucleotide sequence encoding the anti-EBOV GP neutralizing monoclonal antibody (MAb) 6D6 [34], a human–mouse chimeric MAb (ch6D6) was generated. Briefly, the VH and VL coding regions of 6D6 were separately cloned into in-house-developed expression vectors for human IgG1 heavy chain and human kappa light chain, respectively (vector information is strictly restricted under a licensing agreement). The obtained constructs were transfected into Expi293F™ cells (Invitrogen, Carlsbad, CA), and antibodies were purified from the supernatants using Protein A-conjugated Sepharose columns (GE Healthcare, Malmo, Sweden). An anti-2,4-dinitrophenol (DNP) chimeric antibody was prepared similarly as a negative control, using a mouse anti-DNP antibody [35].

### 2.3. Experimental Infection of Rats, Mice, and Hamsters

All animal studies were carried out in strict accordance with the Guidelines for Proper Conduct of Animal Experiments of the Science Council of Japan. The protocol was approved by the Hokkaido University Animal Care and Use Committee. Six-week-old male F344/N rats, 6-week-old male BALB/c mice, and 6- or 3-week-old male Syrian hamsters were purchased from Sankyo Lab Service. All animals were housed in animal biosafety level-2 (BSL-2) or -3 (BSL-3) facilities in the Research Center for Zoonosis Control, Hokkaido University.

For the initial experiment, 6 rats, 6 mice, and 6 hamsters were inoculated intraperitoneally (i.p.) with rVSV/EBOV (10^7^, 10^6.5^, and 10^7.2^ PFU, respectively) in a total volume of 300, 100, and 500 μL, respectively, and 3 animals from each group were euthanized on 2 days post-infection (dpi) for tissue sampling, and the remaining 3 animals were weighed daily for 8 dpi. Surviving animals were euthanized at 8 dpi. and serum samples were collected for antibody detection. To determine the 50% lethal dose (LD_50_), four groups of 6-week-old male Syrian hamsters were infected i.p. with rVSV/EBOV (10^3^–10^6^ PFU) or rVSV/VSV (10^3^–10^6^ PFU) in a total volume of 200 μL and monitored for 7 dpi. Three groups of three 6-week-old Syrian hamsters were also infected i.p. with rVSV/MARV (10^5.5^–10^7.5^ PFU) in a total volume of 500 μL and monitored for 7 dpi. To investigate virus replication in hamsters, each group (of four 3-week-old hamsters) was infected i.p. with 10^7^ PFU of rVSV/EBOV, rVSV/MARV, or rVSV/VSV in a total volume of 1000 μL. All surviving animals were euthanized 36 h after infection and tissue samples were collected from euthanized and deceased animals for virus titration and histopathological analysis. To analyze the changes in blood cell counts and blood chemistry, each group (of four 3-week-old hamsters) was infected i.p. with 10^7^ PFU of rVSV/EBOV, rVSV/MARV, or rVSV/VSV in a total volume of 1000 μL. All animals were euthanized 12 h after infection and blood samples were collected.

### 2.4. Virus Titration

Virus titers were determined by plaque assays. Briefly, confluent Vero E6 cells in 12-well plates were inoculated with 10-fold serial dilutions of 10% tissue homogenates and incubated for 1 h at 37 °C with 5% CO_2_. After the inoculum was removed, the cells were washed with DMEM and overlaid with Eagle’s minimum essential medium containing 0.8% Bacto Agar (Becton Dickinson). After 48 h of incubation, cells were fixed with 10% formalin and stained with crystal violet. Virus titers were determined as PFU.

### 2.5. Histology and Immunohistochemistry

Liver, spleen, kidney, heart, lung, brain, salivary gland, testis, and colon samples were fixed in 10% neutral buffered formalin. The samples were trimmed, embedded in paraffin, sectioned at 5 μm with a microtome, placed on glass slides, and stained with hematoxylin and eosin (HE). The presence of viral antigens in tissues was also investigated by a conventional immunohistochemistry (IHC) technique using HE-unstained sections. Slides were deparaffined in xylene and rehydrated through a series of graded ethanol. Endogenous peroxidase activity was blocked by incubation in 3% hydrogen peroxide in methanol for 10 min. The sections were washed twice in phosphate-buffered saline (PBS) and incubated with 10% normal goat serum (Histofine SAB-PO (R) kit, Nichirei Corporation) for 30 min. Then, they were exposed overnight to a rabbit anti-VSV N polyclonal antibody (in house) at a dilution of 1:1000 at 4 °C. After washing with PBS, a biotinylated goat antibody to rabbit immunoglobulin (Histofine SAB-PO (R) kit, Nichirei Corporation) was applied, followed by incubation at room temperature for 60 min. The immunohistochemical reactions were developed in freshly prepared 3,3’-diamino-benzidine tetrahydrochloride (Histofine SAB-PO (R) kit, Nichirei Corporation). Slides were counterstained with hematoxylin and coverslipped in a mounting medium.

### 2.6. Hematology and Blood Biochemistry

Whole blood samples collected in EDTA tubes were used for standard hematologic analysis using VETSCAN HM2 (ABAXIS) according to the manufacturer’s instructions. The numbers of the following cell types were counted: white blood cells (WBC), red blood cells (RBC), platelets (PLT), monocytes (MON), lymphocytes (LYM), and granulocytes (GRA). Blood biochemistry analysis was performed with VETSCAN VS2 (ABAXIS). The following parameters were measured: alanine aminotransferase (ALT), aspartate aminotransferase (AST), and alkaline phosphatase (ALP).

### 2.7. Passive Immunization of Hamsters with Neutralizing Antibody ch6D6

Three-week-old Syrian hamsters (Sankyo Lab Service) were treated i.p. with 100 μg of MAb ch6D6 one day before or one hour after virus challenge with rVSV/EBOV (10^7^ PFU), rVSV/MARV (10^7^ PFU), or rVSV/VSV (10^7^ PFU). An irrelevant Mab, chDNP, was used as a negative control antibody. The hamsters were monitored for signs of illness and their body weights were measured daily for 8 days. The animals were euthanized once they showed neurological signs such as paralysis and/or more than 20% body weight loss based on their baseline measurement at the time of virus inoculation.

### 2.8. Statistical Analysis

All data were analyzed using GraphPad Prism v6.0 software. To assess the whole blood counts and liver parameter changes, we performed a one-way repeated-measures analysis of variance (ANOVA), followed by multiple *t*-tests comparing the average numbers of blood counts and liver parameters using the Tukey method. P values less than 0.05 were considered to be statistically significant.

## 3. Results

### 3.1. Different Susceptibility to rVSV/EBOV among Rodents

**Figure 1:**
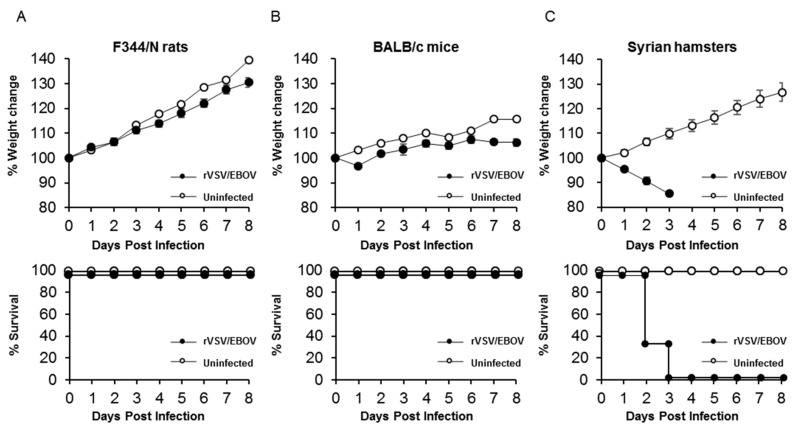
Body weight changes and survival curves of animals after rVSV/EBOV infection. (**A**) F344/N rats, (**B**) BALB/c mice, and (**C**) Syrian hamsters (3 animals for each group) were injected intraperitoneally (i.p.) with 10^7^, 10^6.5^, and 10^7.2^ PFU of rVSV/EBOV, respectively. The symbols represent mean group weights and the bars represent standard errors.

We first compared the susceptibilities of F344/N rats, BALB/c mice, and Syrian hamsters to rVSV/EBOV. Groups of three rats, mice, and hamsters were inoculated with high doses of rVSV/EBOV (approximately 10^6^–10^7^ PFU) via i.p. injection and monitored for signs of illness and body weight changes after infection. All of the rats and mice survived up to the end of the experiment and none of them showed body weight loss (Figure 1A,B). Using an enzyme-linked immunosorbent assay (ELISA), we detected high titers (>10,000) of EBOV GP-specific IgG antibodies in serum samples collected from the surviving animals (data not shown), indicating that these animals were asymptomatically infected with the virus. In contrast, rVSV/EBOV-infected hamsters showed significant body weight loss and succumbed at 2 or 3 dpi (Figure 1C). These data indicated that rats, mice, and hamsters were susceptible to rVSV/EBOV, but that the virus had the potential to cause lethal disease only in hamsters.

### 3.2. Lethal Infection with Recombinant VSVs in Syrian Hamsters

**Figure 2:**
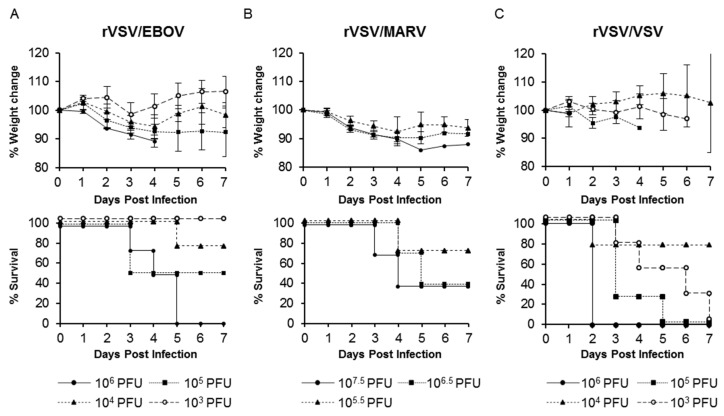
Body weight changes and survival curves of Syrian hamsters infected with rVSV/EBOV, rVSV/MARV, or rVSV/VSV. (**A**) Four hamsters were infected i.p. with 10^6^, 10^5^, 10^4^, or 10^3^ PFU of rVSV/EBOV. (**B**) Three hamsters were infected i.p. with 10^7.5^, 10^6.5^, or 10^5.5^ PFU of rVSV/MARV. (**C**) Four hamsters were infected i.p. with 10^6^, 10^5^, 10^4^, or 10^3^ PFU of rVSV/VSV. All animals were monitored for body weight and survival for 7 days post-infection (dpi). The symbols represent mean group weights and the bars represent standard errors (**A**, **B**, and **C** upper panel).

To determine the LD_50_ of rVSV pseudotyped with the GP of EBOV or MARV (another human-pathogenic filovirus), Syrian hamsters were infected with rVSV/EBOV or rVSV/MARV. The parental VSV (rVSV/VSV) was also tested to determine its pathogenicity in hamsters. Three or four groups of hamsters were infected i.p. with serially diluted rVSV/EBOV, rVSV/MARV, or rVSV/VSV and their body weight loss and clinical signs were monitored. We found that all these viruses induced body weight loss and fatal outcomes (Figure 2). Among rVSV/EBOV-infected hamsters (Figure 2A), animals infected with 10^6^ PFU of the virus showed the most severe body weight loss and all of them succumbed within 5 dpi. The severity of body weight reduction was virus dose-dependent and the calculated LD_50_ value was 10^4.8^ PFU. All hamsters infected with rVSV/MARV also showed a significant body weight loss in a virus dose-dependent manner (Figure 2B). Two of three animals infected with 10^6.5^ and 10^7.5^ PFU of rVSV/MARV succumbed within 4 dpi. (LD_50_ = 10^6.2^ PFU) (Figure 2B). rVSV/VSV also caused severe and fatal diseases in hamsters as described previously [36,37], and the LD_50_ value of rVSV/VSV was lower (10^2.8^ PFU) than those of rVSV/EBOV and rVSV/MARV (Figure 2C).

### 3.3. Virus Dissemination and Pathological Change in Recombinant VSV-Infected Syrian Hamsters

**Figure 3:**
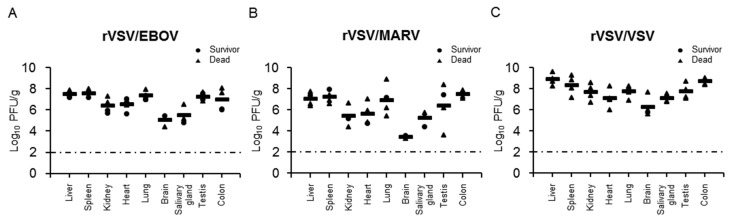
Virus titers in hamsters infected with rVSVs. Four hamsters in each group were infected i.p. with 10^7^ PFU of (**A**) rVSV/EBOV, (**B**) rVSV/MARV, or (**C**) rVSV/VSV. Thirty-six hours after virus inoculation, tissue samples were collected and used for virus titration in plaque assays. Two of four rVSV/EBOV-infected, three of four rVSV/MARV-infected, and three of four rVSV/VSV-infected animals already succumbed at the sampling time point. Each symbol represents the value of an individual hamster. The bars represent the means for the infected hamsters. The broken lines indicate the detection limit (<2.0 log_10_ PFU/g).

**Figure 4:**
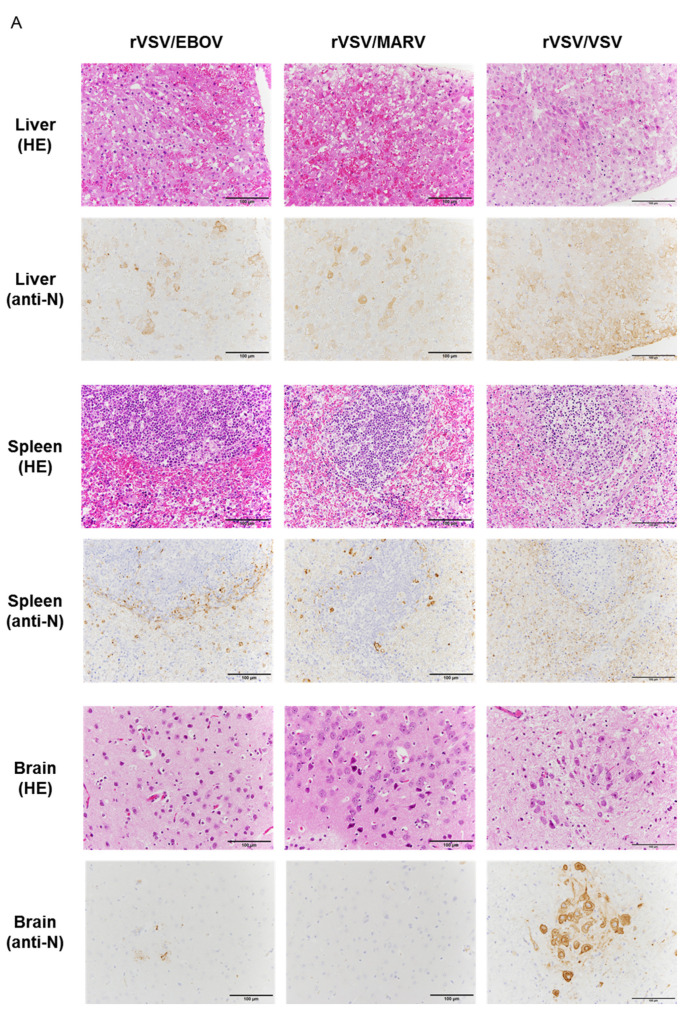

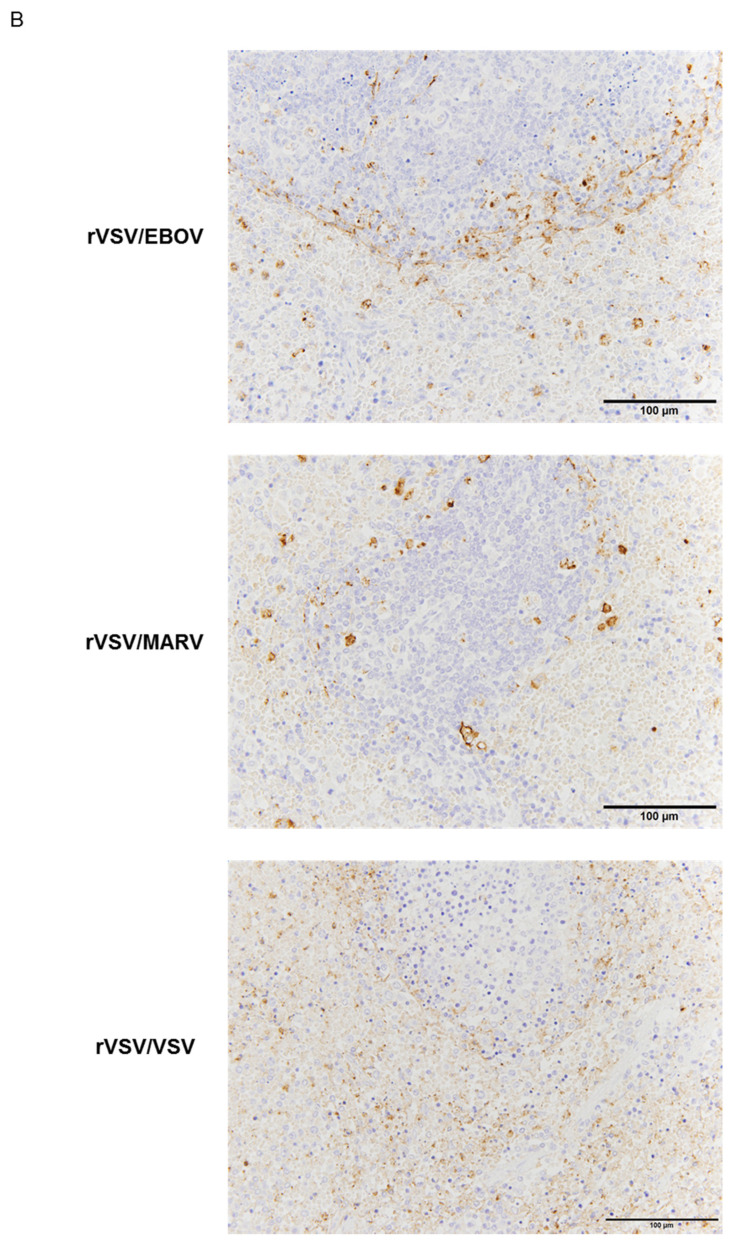
Histological and immunohistochemical analyses of liver, spleen, and brain (cerebral cortex). (**A**) Tissue sections were stained with hematoxylin and eosin (HE) and a rabbit anti-VSV N polyclonal antibody. (**B**) Enlarged images of the spleen sections stained with the anti-VSV N antibody are also shown. Scale bars represent 100 μm. Brown-stained cells represent viral antigen-positive cells.

We then investigated viral replication in hamsters (Figure 3). Three groups of four Syrian hamsters were infected with rVSV/EBOV, rVSV/MARV, or rVSV/VSV and tissue samples (liver, spleen, kidney, heart, lung, brain, salivary gland, testis, and colon) were collected 36 h after infection to determine viral titers (Figure 3) and to examine pathological changes (Figure 4). All the tested tissue samples had detectable viral titers and the mean viral loads ranged from 10^4.30^ to 10^7.53^, 10^2.70^ to 10^7.45^, and 10^6.27^ to 10^8.88^ PFU per gram of tissue for rVSV/EBOV, rVSV/MARV, and rVSV/VSV, respectively. In rVSV/EBOV-infected hamsters, the infectious virus was recovered from liver, spleen, lung, testis, and colon tissues at high titers and relatively lower titers of the virus were detected in the kidney, heart, brain, and salivary gland (Figure 3A). The viral load in brain tissue was the lowest (10^5.12^ PFU/g). Similar trends were observed in rVSV/MARV-infected hamsters (Figure 3B). The virus titers in the brain were remarkably low (10^3.12^ PFU/g). Compared with rVSV/EBOV and rVSV/MARV, viral titers of rVSV/VSV were overall higher in all the tissue samples, but the difference among tissues was less prominent (Figure 3C). These results indicated that rVSV/EBOV, rVSV/MARV, and rVSV/VSV caused systemic infection of hamsters, but that the viral tropisms differed among the viruses.

Viral antigens were then investigated by IHC using the rabbit anti-VSV N polyclonal antibody (Figure 4A). In the liver, the viral antigen was detected in hepatocytes and capillary vessels along with severe hepatocellular necrosis and mild inflammation uniformly in rVSV/VSV-, rVSV/EBOV-, and rVSV/MARV-infected hamsters. In the spleen, viral antigen-positive cells were mainly observed around white pulp in rVSV/EBOV- and rVSV/MARV-infected hamsters (Figure 4B). In rVSV/EBOV- and rVSV/MARV-infected hamsters, strong immunostaining of the viral antigen was seen in monocytic cells. In contrast, many cell types including monocytic cells and lymphocytes were viral antigen-positive in rVSV/VSV-infected animals (Figure 4B). In the brain, where rVSV/EBOV and rVSV/MARV replicated less efficiently than rVSV/VSV, the viral antigen distribution was different among the viruses; viral antigen-positive neurons were extensively observed in rVSV/VSV-infected hamsters and capillary vessels and neurons were weakly stained in rVSV/EBOV-infected hamsters (Figure 4A). In the lung, capillary cells predominantly showed positive signals of the viral antigen (date not shown). Taken together, the IHC results were consistent with the viral loads in the organs and indicated that viral tropisms and primary target cells varied among the viruses.

### 3.4. Hematology and Blood Biochemistry in Syrian Hamsters Infected with Recombinant VSVs

**Figure 5:**
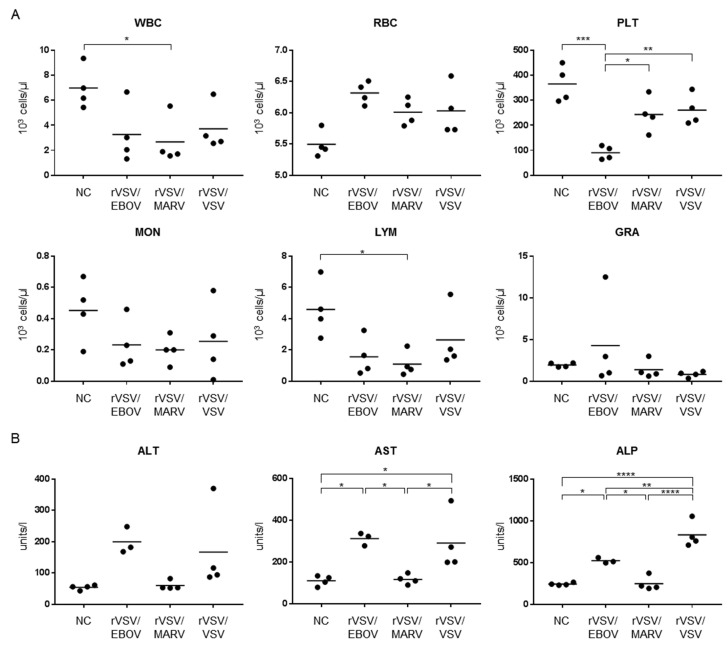
(**A**) Blood cell counts and (**B**) liver parameters in hamsters infected with rVSVs. Four hamsters in each group were infected i.p. with 10^7^ PFU of rVSV/EBOV, rVSV/MARV, or rVSV/VSV. Twelve hours after virus inoculation, blood and serum samples were collected and used for cell counts and blood biochemistry. Each symbol represents the value for an individual hamster. The bars represent the means of the infected hamsters. * *p* < 0.05, ** *p* < 0.005, *** *p* < 0.0005, **** *p* < 0.00005.

To investigate the hematologic and blood biochemical changes, animals infected with rVSV/EBOV, rVSV/MARV, and rVSV/VSV were euthanized 12 h after infection and blood samples were collected for analysis. We observed differences in the WBC, PLT, and LYM counts among rVSV/EBOV-, rVSV/MARV-, and rVSV/VSV-infected hamsters (Figure 5A). The numbers of WBC and LYM decreased upon infection in all the infected hamsters, although a significant difference was only found in rVSV/MARV-infected animals. Interestingly, we observed a significant reduction of the PLT count in rVSV/EBOV-infected hamsters. Although this effect was slightly seen in rVSV/MARV- and rVSV/VSV-infected animals, it was not significant compared to uninfected animals. Significant upregulation of liver parameters, AST and ALP, were observed in rVSV/EBOV- and rVSV/VSV-infected hamsters (Figure 5B). ALT was slightly, but not significantly, increased in rVSV/EBOV- and rVSV/VSV-infected hamsters. These results suggested that viral pathogenesis might differ among rVSV/EBOV, rVSV/MARV, and rVSV/VSV.

### 3.5. Prophylactic and Therapeutic Effects of Antibody Treatment of Syrian Hamsters Infected with rVSV/EBOV

**Figure 6:**
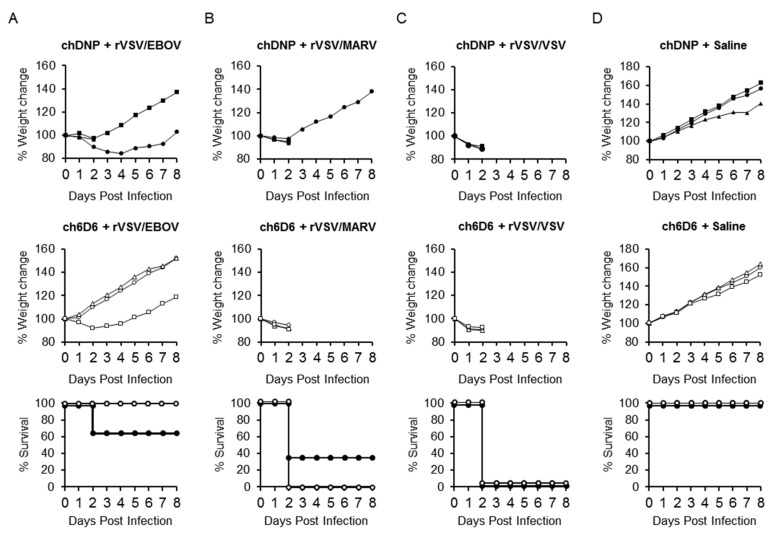
Effects of prophylactic treatment with the neutralizing antibody in rVSV/EBOV-infected hamsters. Three hamsters in each group were treated i.p. with 100 μg of MAb ch6D6 or negative control MAb chDNP one day before (**A**) rVSV/EBOV, (**B**) rVSV/MARV, or (**C**) rVSV/VSV i.p. challenge (10^7^ PFU), or (**D**) treated with saline alone. Symbols represent percentages of body weight for each hamster (upper and middle panels). Open and solid symbols represent survival curves of 6D6- and chDNP-treated animals, respectively (lower panel).

**Figure 7:**
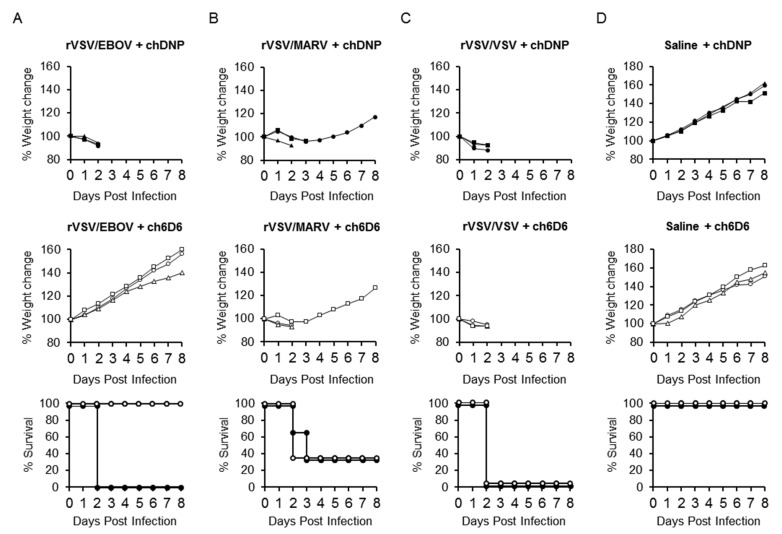
Therapeutic effect of post-exposure treatment with the neutralizing antibody in rVSV/EBOV-infected hamsters. Three hamsters in each group were treated i.p. with 100 μg of MAb ch6D6 or negative control MAb chDNP one hour after (**A**) rVSV/EBOV, (**B**) rVSV/MARV, or (**C**) rVSV/VSV i.p. challenge (10^7^ PFU), or (**D**) treated with saline alone. Symbols represent percentages of weight for each hamster (upper and middle panels). Open and solid symbols represent survival curves of 6D6- and chDNP-treated animals, respectively (lower panel).

To test the utility of rVSV/EBOV-infected Syrian hamsters for in vivo screening of anti-EVD drugs, the prophylactic and therapeutic effects of passive immunization with an anti-EBOV GP-neutralizing MAb were investigated in this model. We used a human-mouse chimeric MAb (ch6D6) generated based on the variable region sequence of mouse MAb 6D6, which has been shown to provide high levels of protection against EBOV infection in mice [34,38]. Syrian hamsters were treated with 100 μg of MAb ch6D6 or chDNP (negative control MAb), one day before (Figure 6) or one hour after (Figure 7) the challenge with rVSV/EBOV, rVSV/MARV, or rVSV/VSV and their body weight changes and survival rates were monitored. The prophylactic treatment of animals with ch6D6 protected all three animals from lethal rVSV/EBOV infection, though one animal showed body weight loss. In contrast, all three animals that received chDNP showed body weight loss and one of them died at 2 dpi. As expected, the treatment of animals with ch6D6 did not show protective effects against rVSV/MARV and rVSV/VSV infection. The post-exposure therapeutic effect was also confirmed in this model (Figure 7). All animals treated with ch6D6 one hour after the virus challenge survived without body weight loss, whereas all of the chDNP-treated animals succumbed at 2 dpi. Infection of animals with rVSV/MARV and rVSV/VSV induced severe disease signs with remarkable weight loss regardless of the treatment and most of them succumbed at 2 or 3 dpi.

## 4. Discussion

In this study, we investigated the pathogenic potential of rVSV/EBOV and rVSV/MARV in mice, rats, and hamsters and evaluated the hamster model as an in vivo GP-targeting drug screening tool that could be handled under BSL-2 conditions. We first inoculated rats and mice with high titers of rVSV/EBOV, but these animals did not show any disease signs and infectious virus was not recovered from any of the tested tissues collected at 2 dpi (data not shown). However, we found that GP-specific serum IgG antibodies were detected at high titers in all of the animals at the end of each experiment (data not shown), indicating asymptomatic infection in rats and mice by rVSV/EBOV. In contrast, rVSV/EBOV infection resulted in fatal outcomes for Syrian hamsters. It was reported that rVSV/EBOV used as a live attenuated vaccine candidate induced strong immunity against EBOV GP and protected 6-week-old hamsters from lethal MA-EBOV infection [29]. In that study, the hamsters were inoculated with 10^5^ PFU of rVSV/EBOV and systemic replication of rVSV/EBOV was confirmed at 1 dpi, but it is unclear whether these rVSV/EBOV-infected hamsters showed disease signs or not. In our study, hamsters infected with 10^5^ PFU of rVSV/EBOV showed significant body weight loss and some of them succumbed within 2 dpi. Infection with a higher dose (10^7^ PFU) of rVSV/EBOV also led to fatal outcomes and the virus was recovered from all the tested tissues, consistent with the previous study [29]. It is conceivable that the difference in susceptibility to VSV among hamster strains affected the different outcomes. Previous reports and our results indicate that wild-type VSV Indiana induces fatal infection of hamsters [36,37,39]. However, differential susceptibility among inbred Syrian hamster strains to the VSV Indiana serotype was reported [36,37]. The susceptibility of outbred Syrian hamsters to rVSV/EBOV might vary depending on their genetic backgrounds.

Interestingly, the tissue tropism of rVSV/EBOV and rVSV/MARV differed from that of rVSV/VSV. In rVSV/VSV-infected animals, the virus was detected in all of the tested organs at high titers, suggesting the pantropicity of this virus. By contrast, rVSV/EBOV and rVSV/MARV seemed to prefer target organs such as the liver, spleen, and lung. The virus titers were much lower in the brain samples collected from rVSV/EBOV- and rVSV/MARV-infected hamsters than in those from rVSV/VSV-infected animals, which is consistent with the observation that the nervous system is not the main target of EBOV and MARV [3]. Importantly, the histopathological analysis of the infected animals also revealed different cellular tropisms among the viruses. It is noteworthy that rVSV/EBOV and rVSV/MARV, but not rVSV/VSV, preferentially infected monocytic cells in the spleen, which are known to be one of the major target cells of EBOV and MARV [40,41]. These observations suggest that rVSV/EBOV and rVSV/MARV might have tissue tropisms similar to those seen in actual EBOV and MARV infection in mice and nonhuman primates. Since inflammation is most likely the key factor of pathology in most animal models and human cases of EBOV and MARV infections, the infection of macrophages and dendritic cells might also play important roles in the pathogenesis of this hamster model although the immune regulation mechanisms mediated by viral proteins other than GP should differ from those in animals infected with authentic EBOV and MARV.

Different viral tropisms are often determined by the interaction between viral envelope proteins and their host receptors. VSV exhibits a robust pantropic infectivity mediated by its envelope protein, VSV G. It has been reported that nonspecific electrostatic and hydrophobic interactions mediate the attachment of VSV to cells [42,43]. The involvement of the cell surface LDL receptor as the major ubiquitous VSV cellular receptor has also been suggested [42,43]. On the other hand, the filovirus GP-mediated attachment to host cells is believed to have tropism to cells expressing C-type lectins such as dendritic cell- and liver/lymph node-specific ICAM-3-grabbing non-integrin (DC/L-SIGN), human macrophage galactose-type C-type lectin (hMGL), and liver and lymph node sinusoidal endothelial cell C-type lectin (LSECtin) [40,41]. Hepatocytes, endothelial cells, dendritic cells, monocytes, and macrophages, all of which express C-type lectins, are thought to be preferred target cells of EBOV and MARV. In this study, we found that rVSV/EBOV and rVSV/MARV infected hepatocytes, macrophages, and Kupffer cells, although tropism to endothelial cells was not clearly observed. In contrast, rVSV/VSV widely infected many types of cells, including hepatocytes, macrophages, lymphocytes, and capillary cells. These results suggest that rVSV/EBOV and rVSV/MARV preferentially infect particular types of cells, which is reflected by GP-mediated cell tropism, likely through the interaction with C-type lectins.

Patients having EVD or MVD typically present with abnormally low numbers of leukocytes, lymphocytes, and platelets at the time of clinical onset [44,45]. In all rVSV-infected animals, leukocytopenia and lymphocytopenia were observed and the degree was most severe in rVSV/MARV-infected hamsters. Interestingly, severe thrombocytopenia was only observed in rVSV/EBOV-infected hamsters. The coagulopathic state seen in EVD appears to be caused by a combination of activation of the mononuclear phagocytic system, platelet aggregation, and consumption [4,44,45]. Our data suggest that rVSV/EBOV-infected hamsters share a similar pathological mechanism with EBOV infection of humans and nonhuman primates, though rVSV/MARV seemed to have lower preference for the mononuclear phagocytic system.

Significant increases of ALT and AST and a high AST/ALT ratio are common features of EVD and MVD, indicating acute hepatic failure [44,45]. In the present study, elevated levels of serum liver enzymes were observed in rVSV/EBOV-infected hamsters, but not in rVSV/MARV. We assumed that rVSV/EBOV induced pathological changes similar to those in actual EBOV infection of humans and nonhuman primates, whereas rVSV/MARV caused milder infection in hamsters, resulting in less prominent pathological changes. The increase of the liver enzymes was also seen in rVSV/VSV-hamsters. Considering the pantropic property of VSV, elevated levels of the enzymes in rVSV/VSV-infected hamsters, particularly ALP levels that were higher than in rVSV/EBOV-infected animals, might suggest that rVSV/VSV induced severe multiple organ disfunction that was distinct from that observed in rVSV/EBOV- and rVSV/MARV-infected animals.

In the present study, we confirmed the pathogenicity of replication-competent rVSV pseudotyped with filovirus GPs in Syrian hamsters. Although the detailed pathogenesis of the disease should be different from EBOV and MARV infection in humans, we demonstrated the utility of this model to investigate the prophylactic and therapeutic effects of passive immunization with an anti-GP neutralizing antibody. Since the entry of rVSV/EBOV and rVSV/MARV into target cells depends on the function of GPs, this animal model is a useful tool for screening of anti-GP drugs under BSL-2 conditions and is expected to accelerate drug development targeting the function of GPs.
